# Supplementary material for: The Long-Term Pannexin 1 Ablation Produces Structural and Functional Modifications in Hippocampal Neurons
Source: Cells. 2022 Nov 17;11(22):3646. doi: 10.3390/cells11223646 (PMC9688914; doi:10.3390/cells11223646)
Supplement: Supplementary file 1 [file cells-11-03646-s001.zip › cells-1926928-supplementary.pdf]

cells

MDPI

Supplementary Information

Table S1 Morphological classification of synapses

	Compound synapses[1.]			Type of compound synapses[2]			
	Simple	Double	Triple	I	II	III	IV
WT	80.9±23.3	9.8±1.5	0.80±0.2	2.5±0,6	1.8±0.2	1.5±0.3	0.0±0.0
Panx1-KO	208.0±17.6	58.2±3.3	4.4±0.9	4.0±0.4	15±1.6	6.8±0.8	2.3±0.5

Data are mean ± SEM. *Statistical significance determined by 2way ANOVA (p<0.05) compared to WT group. [1]Number of synapses. Compound synapses refer to double and triple synapses. [2]Type I = axon with one spine; Type II= axon with two contacts on the same spine; Type III= axon with two spines; Type IV= axon with three or more contacts.

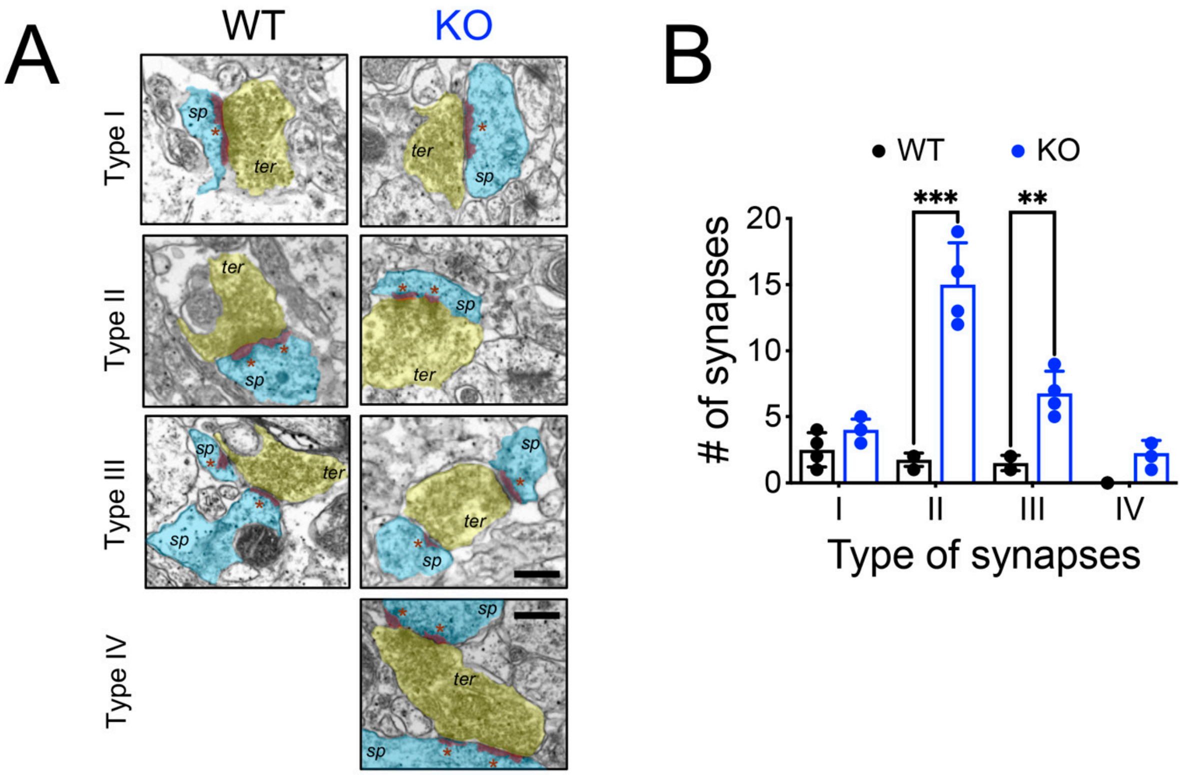

Figure S1. Morphological classification of hippocampal synapses**.** Representative transmission electron microscopy photographs of asymmetric synapses (**A**) and analysis of the types of synapses (**B**) in CA1 *Stratum radiatum* area of WT (black) and PANX1-KO (KO, blue) mice. Magnification 43.000X, bar: 500 nm. Yellow area represents presynaptic terminals, blue area represents dendritic spines, and red area represents PSD. Red asterisk indicates synaptic contact. $n = 6$ (WT) and $n = 6$ (KO) ultrathin sections from 3 animals, **p=0.002, ***p<0.001 2way ANOVA test.

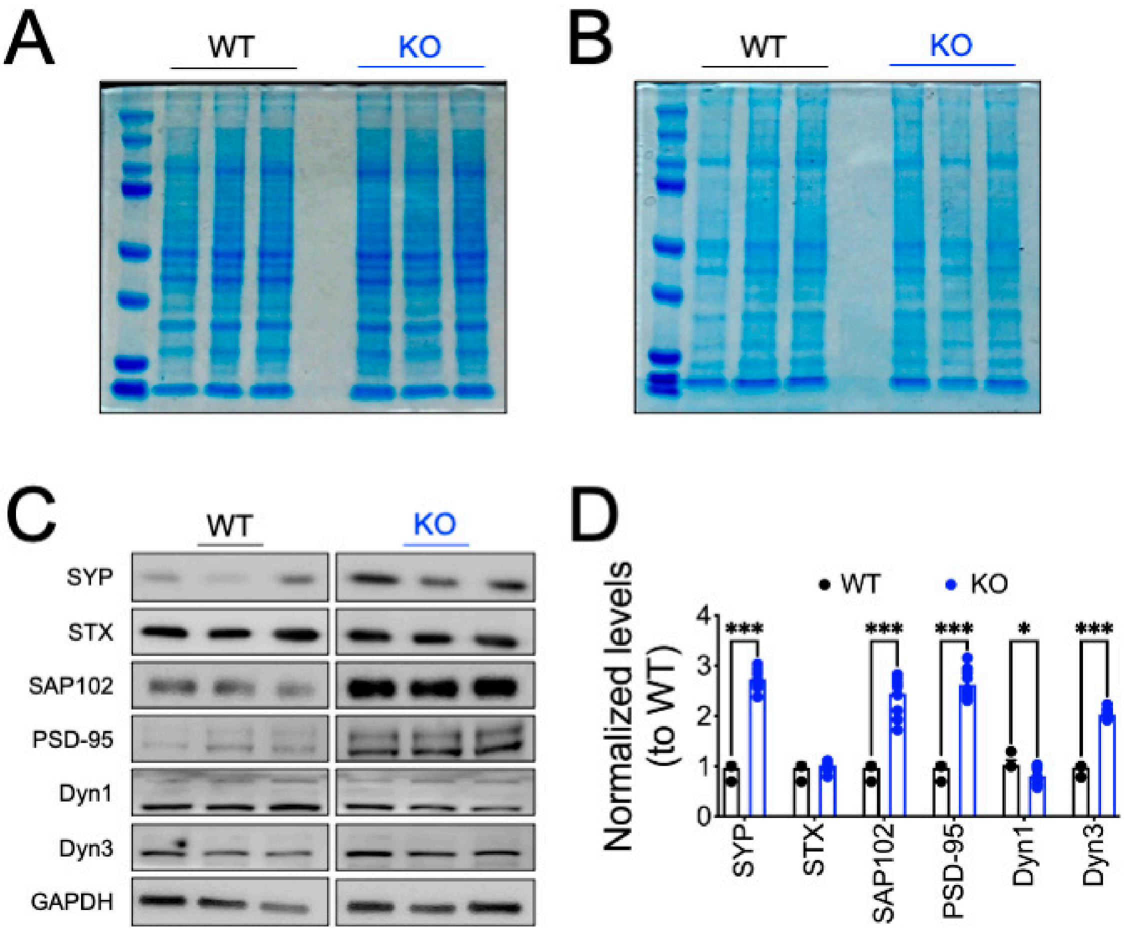

Figure S2. Synaptic proteins levels of hippocampal tissue. Representative images of SDS-PAGE and Coomassie blue-stained gels of hippocampal extracts from WT (black) and PANX1-KO (KO, blue) mice. (**A**) and (**B**) PSD-enriched fractions. (**C**) Representative blots and densitometric analysis of hippocampal synaptic proteins (**D**) $n = 6$ (WT) and $n = 6$ (KO) slices from 6 animals, *p=0.0260, ***p<0.001 2way ANOVA test.

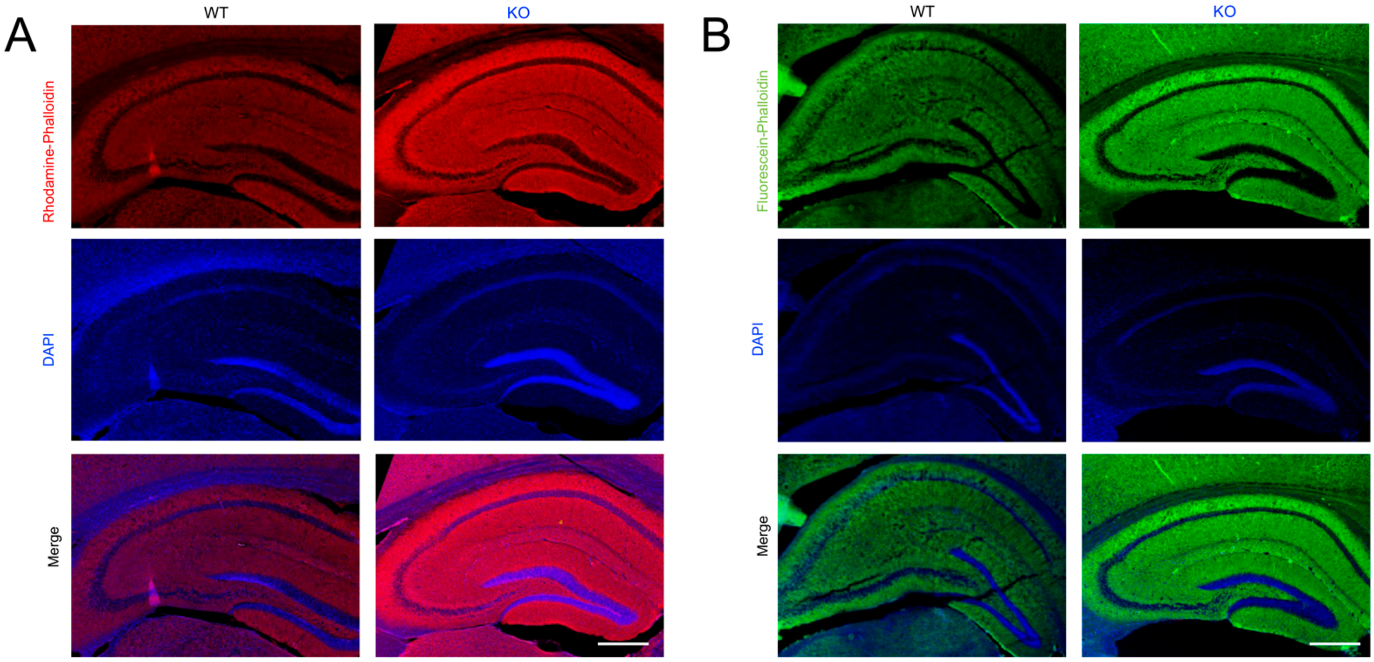

Figure S3. Phalloidin labeling of actin filaments in hippocampal tissue from WT and Panx1-KO brains. A, Representative images showing the rhodamine-phalloidin (red) and DAPI reactivity in the CA1 region of the hippocampus. B, Representative images showing the fluorescein-phalloidin (green) and DAPI reactivity in the CA1 region of the hippocampus Whole hippocampal at 4X magnification (top panel), scale bar: 150 µm.
